# Analog-sensitive cell line identifies cellular substrates of CDK9

**DOI:** 10.18632/oncotarget.27334

**Published:** 2019-12-10

**Authors:** Tim-Michael Decker, Ignasi Forné, Tobias Straub, Hesham Elsaman, Guoli Ma, Nilay Shah, Axel Imhof, Dirk Eick

**Affiliations:** ^1^Department of Molecular Epigenetics, Helmholtz Center Munich and Center for Integrated Protein Science Munich (CIPSM), Germany; ^2^Biomedical Center Munich, ZFP, Ludwig-Maximilian University Munich, Germany; ^3^Bioinformatic Unit, Biomedical Center Munich, Ludwig-Maximilian University Munich, Planegg-Martinsried, Germany; ^4^Present address: Department of Biochemistry, University of Colorado, Boulder, USA; ^5^Present address: Stowers Institute for Medical Research, Kansas City, Missouri, USA

**Keywords:** CDK9, protein kinase, transcription, RNA splicing, phosphoproteomics

## Abstract

Transcriptional cyclin-dependent kinases regulate all phases of transcription. Cyclin-dependent kinase 9 (CDK9) has been implicated in the regulation of promoter-proximal pausing of RNA polymerase II and more recently in transcription termination. Study of the substrates of CDK9 has mostly been limited to *in vitro* approaches that lack a quantitative assessment of CDK9 activity. Here we analyzed the cellular phosphoproteome upon inhibition of CDK9 by combining analog-sensitive kinase technology with quantitative phosphoproteomics in Raji B-cells. Our analysis revealed the activity of CDK9 on 1102 phosphosites quantitatively, and we identified 120 potential cellular substrates. Furthermore, a substantial number of CDK9 substrates were described as splicing factors, highlighting the role of CDK9 in transcription-coupled splicing events. Based on comparison to *in vitro* data, our findings suggest that cellular context fundamentally impacts the activity of CDK9 and specific selection of its substrates.

## INTRODUCTION

Phosphorylation by protein kinases is a major post-translational modification in cell signaling. Higher eukaryotes encode for 518 putative protein kinases and many of them are expressed in cells at the same time [1]. While there are hundreds of kinases, only three amino acids, serine, threonine, and tyrosine, undergo modification by kinases in eukaryotes [2]. Analysis of the sequence and structure of phosphorylation sites has revealed only limited specificity *in vivo* and *in vitro* [3]. Given the large number of kinases and their limited specificity, protein phosphorylation apparently undergoes several layers of regulation. Recruitment of kinases and control of their activity substantially contribute to the regulation of protein phosphorylation *in vivo* [4].

The question of the number of kinases that can participate in phosphorylation of a target site *in vivo* is difficult to answer. Kinases can be removed by genetic knockout or by RNA interference-mediated downregulation. Alternatively, the activity of kinases can be inhibited by chemical inhibitors of varying specificity [5]. Notably, such inhibitors are of high therapeutical interest, as many kinases are involved in human cancer [6]. However, all these approaches usually do not represent a direct proof for the phosphorylation of a substrate by a specific kinase *in vivo*.

Cyclin-dependent kinase 9 (CDK9) is a central regulator of eukaryotic transcription and a promising therapeutical target in cancer and other diseases [7]. In mammals, CDK9 is the kinase subunit of Positive Transcription Elongation Factor b (P-TEFb). Its role in releasing promoter-proximal paused RNA Polymerase II (Pol II) is well established, and several substrates of CDK9 in this process have been described previously: Pol II at its C-terminal repeat domain (CTD), negative elongation factor NELF, and DSIF which promotes transcription elongation upon phosphorylation [8–10]. Recent *in vitro* studies identified multiple novel substrates of CDK9 and previously unknown phospho-acceptor sites [11, 12]. However, such approaches cannot provide information about the activity of CDK9 in a cellular context.

We have recently created a human B cell line expressing an analog-sensitive CDK9 (CDK9as). These cells are homozygous for F103A mutations at CDK9 gene loci, which renders them sensitive to inhibition by a specific adenine analog. Using this cell line, we previously studied the effects of CDK9 inhibition in cells and demonstrated that CDK9 stimulates release of paused polymerase and activates transcription by increasing the number of transcribing polymerases [13]. Here, we combined this analog-sensitive cell line with phosphoproteomics to study the cellular substrates of CDK9 in a quantitative way.

## RESULTS

### Analog-sensitive CDK9 cells allow for quantitative phosphoproteomics

**Figure 1:**
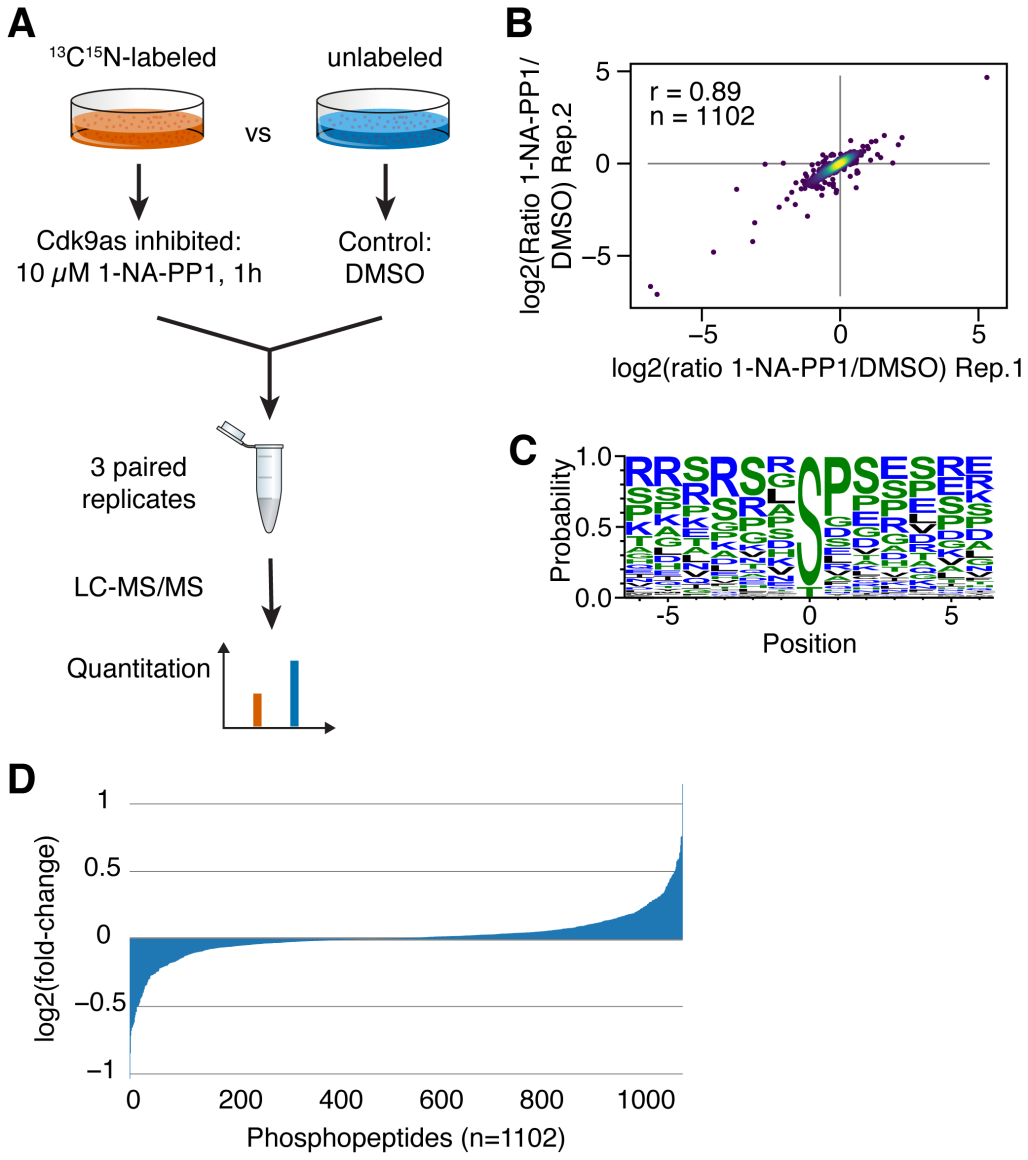
(**A**) Quantitative phosphoproteomics: Inhibition of analog-sensitive CDK9 was combined with SILAC mass spectrometry to quantitate protein phosphorylation. (**B**) Correlation of 1NA-PP1/DMSO ratios among replicates was determined (r = Spearman correlation coefficient). (**C**) Phosphosite motif analysis was performed with Weblogo. (**D**) Distribution of changes in phosphorylation of detected phosphopeptides.

CDK9as cells were recently used to study the effects of CDK9 inhibition on nascent transcription in cells [13]. Here, we utilized this cell line to study substrates of CDK9 in a cellular context and quantitate the contribution of CDK9 to individual phosphosites (Supplementary Figure 1A). First, we analyzed RNA Pol II phospho-CTD levels at different time points of 1-NA-PP1 treatment by western blot (Supplementary Figure 1B). Reduction of phosphorylation levels was weak after 15 min but very robust after 2 h of inhibition. Thus, we next decided to treat CDK9as with 1-NA-PP1 for one hour followed by quantitative phospho-proteomics using SILAC (Figure 1A). Three paired replicates were analyzed and 1102 common phosphosites were detected. Phosphosites showed strong correlation among all replicates and Pearson correlation coefficients ranged from r = 0.71 to r = 0.89 (Figure 1B and Supplementary Figure 2). We identified 120 phosphosites as significantly decreased (*p*-value < 0.05) upon inhibition of CDK9 whereas 172 were increased (data supplement, Supplementary Table 1). Sequence motif analysis of decreased phosphorylations revealed a strong preference for serine as phospho-acceptor (92%) and a moderate preference for proline in the +1 position (45%) (Figure 1C).

Our quantitative phosphoproteomics approach allows for precise measurement of minor changes in the phosphorylation steady state of a given phosphosite upon inhibition of CDK9. Thus, we next sorted all significantly (*p*-value < 0.05) affected phosphosites by fold-change to identify those cellular substrates that were most affected by inhibition of CDK9. Interestingly, most phosphosites were only moderately affected with log2 fold changes below 0.5 (Figure 1D). However, there were several substantially reduced phosphosites (Table 1), indicating that CDK9 considerably contributes to phosphorylation levels of some proteins and less to others, including sites with low turnover of phosphorylation. Together, these findings demonstrate that we can determine even minor changes in the CDK9 phosphoproteome quantitatively. Furthermore, this allows for a deeper analysis of proteins with significantly decreased phosphorylation which will be called CDK9 substrates, hereafter.

**Table 1:** Top 30 reduced CDK9 substrates, ranked by log2(fold-change)

Name	Uniprot	Phosphosite	Log2(FC)	*P*-value
FUNDC2	Q9BWH2	S151	–1.04	4.96E-07
U2SURP	E7ET15	S945, S947, S950	–0.85	3.89E-05
NFATC2	Q13469	S757, S759	–0.71	0.000382
LBR	C9JXK0	S97, S99	–0.68	0.000298
SRSF9	Q13242	S211, S216	–0.66	0.000685
MARCKSL1	P49006	S101, S104	–0.66	0.00191
C18orf25	K7EQH1	S66	–0.63	0.0363
PDCD4	Q53EL6	S76	–0.62	0.00634
NCBP1	F2Z2T1	S7	–0.58	0.00656
BCLAF1	E9PK09	T257, S268	–0.56	6.37E-06
STX7	O15400	S126, S129	–0.54	0.0104
TRIM28	Q13263	S473	–0.5	7.99E-05
MED1	Q15648	S1192	–0.5	0.000182
RHBDF2	Q6PJF5	S325, S328	–0.5	0.000814
RPS6KA1	Q15418	T359, S363	–0.5	0.0364
WDCP	Q9H6R7	S690	–0.48	0.00544
RANBP2	P49792	S1160	–0.44	1.26E-05
YBX1	P67809	S165	–0.43	4.21E-05
SRRM2	Q9UQ35	S1443, S1444	–0.4	0.00184
MIER1	Q8N108	S483, S488	–0.4	0.00654
ACLY	P53396	S455	–0.37	0.00519
SCAF11	A0A0A0MTP7	S711, S716	–0.35	0.00198
LAS1L	Q9Y4W2	S560	–0.34	0.000311
LBR	C9JXK0	S99	–0.34	0.000474
PRRC2A	P48634	S761	–0.32	0.0173
SMARCA4	P51532	S695, S699	–0.3	0.000665
CCDC86	Q9H6F5	S58	–0.3	0.00126
DNAJC2	F2Z3H0	S47, S49	–0.27	0.000589
EIF4B	P23588	S445	–0.27	0.00139
FNBP1	B7ZL14	S296	–0.27	0.00727

### Transcription- and splicing-associated factors are the major substrates of CDK9

**Figure 2:**
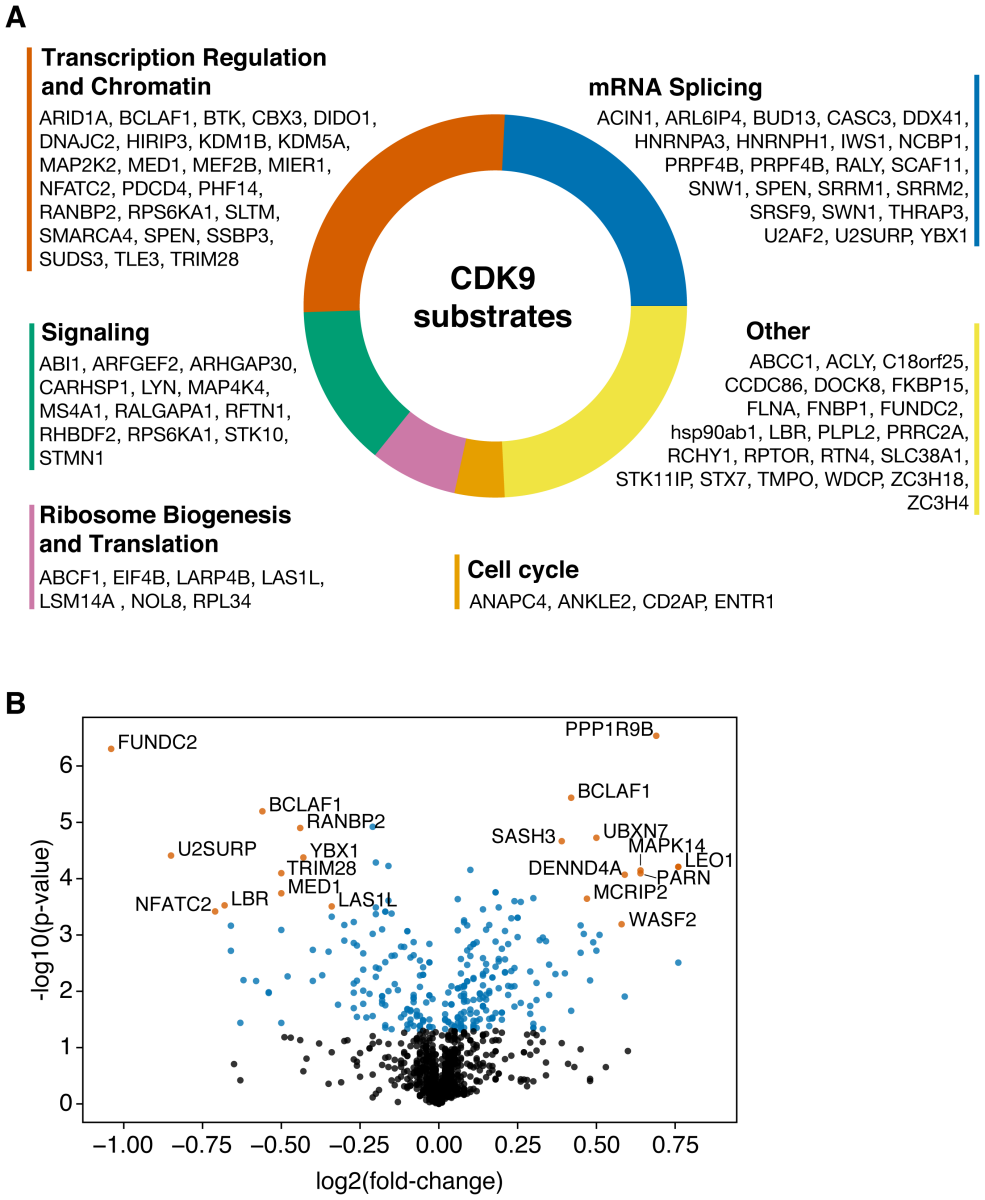
(**A**) Gene ontology analysis of CDK9 substrates. (**B**) Volcano plot showing the CDK9 phosphoproteome. Most significantly up- and downregulated (by *p*-value, log2 fold-change > 0.3) phosphopeptides are annotated.

Due to the well-described role of CDK9 in transcription regulation, we performed gene ontology analysis with CDK9 substrates that were identified in this study. We found 50.5% of CDK9 substrates to be proteins involved in transcription or chromatin regulation and mRNA splicing (Figure 2A and 2B). Interestingly, when weighting substrates by fold change, this majority increased to 58.2%, demonstrating that CDK9 also phosphorylates transcription and splicing-associated factors on a quantity basis (Supplementary Figure 3). Notably, four out of the top ten reduced substrates have no annotated roles in transcription or splicing (FUNDC2, LBR, MARCKSL1, c18orf25). Together, the substrates identified in this study are in line with the role of CDK9 in transcription regulation and further emphasize the role of this kinase in splicing.

### Cellular CDK9 substrates show little overlap with *in vitro* substrates

**Figure 3:**
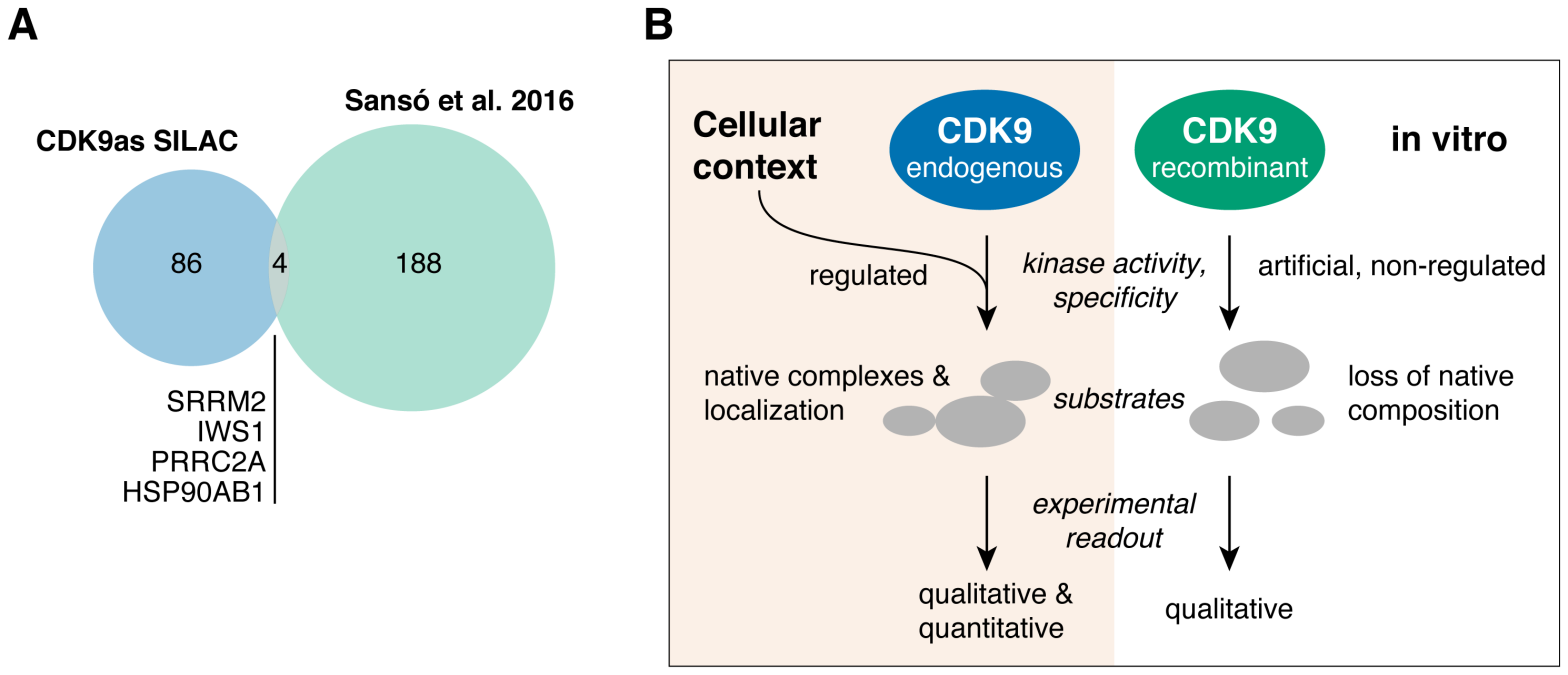
(**A**) Venn diagram depicting the overlap between cellular (this study, CDK9as SILAC) and *in vitro* (11) CDK9 substrates. (**B**) Model: The study of protein kinases and their substrates fundamentally differs when performed outside of cellular context.

Specificity of kinase inhibitors as well as the study of kinase substrates is typically performed *in vitro*. While *in vitro* approaches allow identification of potential CDK9 substrates, they cannot provide information about the activity of CDK9 in cells. Thus, we compared our cellular set of CDK9 substrates to the results of the Fisher lab that determined CDK9 substrates *in vitro* using a combined analog-sensitive and chemical approach [11]. Of 120 cellular substrates, four (HS90B, IWS1, PRRC2A, SRRM2) could be co-identified in the *in vitro* dataset, but only for HS90B we found a matching phosphosite on S255 (Figure 3A). The minimal overlap of cellular and *in vitro* data suggests, that *in vitro* analysis alone limits the understanding of kinases and their inhibitors that can be won in such experiments.

## DISCUSSION

### Quantitative phosphoproteomics puts CDK9 in the center of co-transcriptional events

The canonical role of CDK9 as the kinase subunit of P-TEFb in the release of promoter-proximal pausing of RNA Pol II is well established and has been demonstrated in various studies [8–10]. Surprisingly, our list of CDK9 substrates did not contain several of those substrates, that are mostly linked with the canonical role of CDK9, including Pol II CTD, NELF, and DSIF. This might be explained by the complex nature of our sample, in which peptides of these proteins may be masked by others that are more abundant. Importantly, we did not include any fractionation to enrich for specific proteins in our sample preparation to maintain an unbiased approach, and to specifically identify those phosphopeptides that are quantitatively most important.

Several high confidence substrates identified in the present study are associated with events in early transcription as well, including Mediator subunit MED1 which is part of the pre-initiation complex [14, 15] and TRIM28 (KAP1) which was recently found to recruit the inactive P-TEFb (7SK snRNP) complex [16]. We also detected elongation factor IWS1, although its phosphorylation was only moderately reduced. Notably, IWS1 was also identified as a CDK9 substrate *in vitro* [11], validating this factor as a high confidence substrate of CDK9. IWS1 is recruited to elongating Pol II by SPT6 and promotes mRNA export and recruitment of histone methyltransferase Setd1 [17, 18]. Recent work demonstrated a link between CDK9 and gene silencing at heterochromatic loci and reported SMARCA4 (BRG1) as a direct phosphorylation substrate of CDK9 [19]. We also identified SMARCA4 as a CDK9 substrate, although the phosphosites differ from those detected in the mentioned study. This expands the spectrum of CDK9 substrates by several factors that are central to transcription initiation and elongation.

Intriguingly, we identified a substantial number of additional cellular CDK9 substrates that have a role in mRNA splicing. Indeed, the second most downregulated phosphoprotein in our dataset was U2SURP, which is part of the spliceosome and together with RBM17 and CHERP forms a minimal module that binds the U2 snRNP [20]. Moreover, SRSF9 (SRp30c), a SR protein and regulator of alternative splicing [21, 22], also showed substantially reduced phosphorylation in our dataset. Importantly, alternative splicing was recently found to be increased in tumors [23] and changes in alternative splicing may drive functional transformations in cancer [24]. The coupling of transcription with splicing events is well established [25]. However, the role of individual factors and mechanisms involved are poorly described. P-TEFb has been linked to regulation of splicing before. In a functional proteomics approach, the CDK9 interactome was found enriched for splicing factors, suggesting that these factors are part of the basal CDK9 complex [26]. Furthermore, CDK9 and Cyclin T1 co-localize with splicing speckles in HeLa cells [27]. Another study demonstrated the importance of 7SK snRNP integrity for alternative splicing using cellular splicing assays [28]. In the same study, knockdown of 7SK snRNP subunits larp7 or bcdin3 orthologues in zebrafish embryos resulted in aberrant splicing. Vice versa, splicing factors stimulate transcription elongation [29, 30], and splicing inhibition downregulates Pol II CTD phosphorylation at serine-2 sites [31], indicating reciprocal regulation of the two mechanisms. Together with the evidence from previous studies, our findings suggest that the kinase activity of CDK9 plays a key role in the regulation of RNA splicing by phosphorylation of splicing factors.

### Quantitative phosphoproteomics identifies potential CDK9 substrates

We found a number of new CDK9 substrates that were substantially impacted upon inhibition of CDK9. In our analysis, the top target identified as FUN14 domain-containing protein 2 (FUNDC2). Little is known about this membrane protein, but mitochondrial FUNDC2 has been reported to support platelet survival via the AKT pathway [32]. It is unclear if and how FUNDC2 connects to the function of CDK9 in transcription, but it has been found in the proteome of the human sperm nucleus [33]. The third most reduced phosphorylation was measured for transcription factor NFATC2 at S757 and S759 residues. However, phosphorylation of more amino-terminal serines were found to regulate nuclear localization and transcriptional activation of NFATC2 in previous studies [34, 35]. Thus, phosphorylation by CDK9 might provide similar functional control in a co-transcriptional way. Particularly, CDK9 may phosphorylate NFATC2 to trigger its re-localization to the cytoplasm, establishing a feedback-loop that links elongation to transcription initiation. Moreover, we found NCBP1, a component of the cap-binding complex (CBC), to be strongly reduced in phosphorylation at S7 in CDK9-inhibited cells. This phosphorylation has been demonstrated to be placed by S6 Kinase B1 (RPS6KB1) and activate the CBC [36]. Interestingly, we found S6 Kinase A1 (RPS6KA1), a member of the same kinase family, to be reduced in phosphosites that positively regulate the activity of S6 Kinase A1. This observation indicates both that CDK9 activates other kinases and that it is involved in RNA-export and translation initiation, further expanding its role as a central regulator of co- and post-transcriptional processes.

### Cellular context substantially determines the activity of CDK9

The use of analog-sensitive kinases reduces the risk of off-target inhibition when using generic kinase inhibitors that often lack desirable specificity [37]. Notably, analog-sensitive CDK12 was recently used to study its cellular substrates by mass spectrometry [38], demonstrating that this approach further expands the understanding of transcriptional kinases. CDK9 substrates identified in our study show little overlap with a recent *in vitro* study that used a similar analog-sensitive kinase strategy in combination with cellular extracts [11]. This discrepancy can partly be explained by lack of sensitivity of our phosphoproteomics strategy. In addition, the differences in cell type (B-cell lymphoma *versus* colon carcinoma) and source of analog-sensitive CDK9 (endogenously expressed *versus* excess of recombinantly purified) may provide further explanation. Notably, Sansó et al. found that 50% of their CDK9 substrates have functions in transcription and RNA metabolism and splicing, which is similar to our findings. However, the observed differences emphasize that *in vitro* methods cannot sufficiently predict the effects of kinase inhibition in a cellular context (Figure 3B). This notion is of particular importance when evaluating novel kinase inhibitors for therapeutic purposes. Importantly, a kinase might act on a substrate *in vitro* but its impact on the cellular steady-state of the same phosphosite might be insignificant. In cells, the amount of CDK9 is limited, its recruitment is tightly coordinated, and its activity is highly regulated. Thus, quantitative cell-based methods such as SILAC combined with phosphoproteomics provide valuable insights into the activity of CDK9 and its impact on the phosphorylation state of its substrates.

## MATERIALS AND METHODS

### CDK9^as^ cell line

CDK9 analog-sensitive Raji B cells (CDK9as) used in this study were described previously [13]. CDK9as are homozygous for F103A mutation at CDK9 gene loci, which were introduced using the CRISPR-Cas9 system.

### Western blot analysis and antibodies

Cells were lysed in 2x laemmli buffer before submission to SDS-PAGE. Proteins were transferred to a nitrocellulose membrane (GE) and unspecific binding of antibodies was blocked by 1 h incubation of the membrane with 5% milk (w/v) in Tris-buffered saline plus 1% Tween-20 (TBS-T). Next, the following primary antibodies were added and incubated overnight at 4°C: alpha-Tubulin (Sigma T9026), Cdk9 (Santa Cruz sc-484), Rpb1 (Pol3/3), CTD phospho-specific antibodies: Ser2-P (3E10), Ser5-P (3E8) [39]. The membrane was washed in TBS-T and incubated for 90 min at room temperature with IRDye-labelled, secondary antibodies against rat/rabbit (680 nm; Invitrogen) and mouse (800 nm; Rockford, Biomol) and imaged using an Odyssey Imaging System (Li-Cor).

### Cell culture and SILAC labeling

Cells were maintained in RPMI1640 without arginine, glutamine, and lysine (Silantes, Munich, Germany) supplemented with 10% dialyzed fetal bovine serum (Silantes), 2 mM L-alanyl-L-glutamine (Thermo Fisher Scientific), 100 U/mL penicillin and 100 µg/mL streptomycin (Thermo Fisher Scientific), and 100 µM sodium pyruvate (Thermo Fisher Scientific) at 37°C and 5% CO_2_. For stable isotope labeling with amino acids in cell culture (SILAC), either ^13^C^15^N-labeled or unlabeled L-arginine (40 mg/L) and L-lysine (200 mg/L) (Silantes) were added. Cells were labeled for 12 days prior to CDK9 inhibition assays. Labelling efficiency of CDK9as was validated by mass spectrometry analysis periodically.

### Inhibition of analog-sensitive CDK9

1 × 10^7^ cells were treated with 10 µM 1-NA-PP1 (Merck Millipore) or DMSO (Sigma-Aldrich) for 1 hour. Cells were pelleted by centrifugation and washed once in phosphate buffered saline (PBS). 1-NA-PP1-treated cells and DMSO control cells were pooled resulting in 2 × 10^7^ total cells per replicate. Four biological replicates of labeled, 1-NA-PP1-treated cells pooled with unlabeled, DMSO-treated cells were prepared. In addition, four corresponding label-swapped replicates were prepared, in which unlabeled cells were treated with 1-NA-PP1 and DMSO control cells were labeled with ^13^C^15^N-containing arginine and lysine, resulting in 4 paired replicates.

### Cell lysis and phosphopeptide enrichment

Subsequent sample preparation was essentially based on the EasyPhos method [40] with modifications as follows. Pooled cells were washed once in PBS, solubilized in 400 µL lysis buffer (4% SDS, 100 mM tris pH 8.5, 10 mM tris(2-carboxyethyl)-phosphine (TCEP, Sigma-Aldrich), 40 mM chloroacetamide (CAA, Sigma-Aldrich) and heated at 95°C for 5 min. Lysates were cooled on ice, sonicated (Branson Sonifier 250, 3x 10 pulses, output 5, duty cycle 50%) and heated again at 95°C for 5 min. Lysates were once more cooled on ice and processed on a Bioruptor UCD-200 (Diagenode, 15x [30 sec ON + 30 sec OFF] at high setting). Lysates were centrifuged for 30 min at 3,500 g at 4°C. The supernatant was transferred into a clean tube and protein concentration was determined by BCA assay (Pierce Microplate BCA Protein Assay Kit - Reducing Agent Compatible).

Samples were normalized to a protein concentration of 5 mg/mL and protein was precipitated over night by adding 4x volume of –20°C acetone. Precipitated protein was collected by centrifugation (15 min at 2,000 g) and washed twice in –20°C acetone and by resuspension in the Bioruptor (20 sec, high). Protein pellets were resuspended in 500 µL TFE digestion buffer (10% 2,2,2-trifluoroethanol [Sigma-Aldrich], 100 mM ammonium bicarbonate). Protein digest and phosphopeptide enrichment were performed as described in Humphrey *et al.*, 2015. Protein digest was performed using Trypsin and LysC. For phosphopeptide enrichment Titansphere Phos-TiO Beads 10 µm were used (GL Sciences). Eluted phosphopeptides were concentrated by speed-vac and brought to 3% TFA.

### Desalting and LC-MS/MS

For desalting, peptides were loaded onto 2x layers styrenedivinylbenzene-reversed phase-sulfonated StageTips (SDB-RPS; 3M Empore) and washed 1) in 100 μL SDB-RPS wash buffer 1 (99% isopropanol, 1% trifluroacetic acid [TFA]) and 2) in 100 µL SDB-RPS wash buffer 2 (0.2% TFA). Phosphopeptides were eluted with 60 μL SDB-RPS elution buffer (80% acetonitrile, 1.25% NH_4_OH [25%, HPLC grade]) into a clean protein low-binding tube by centrifugation for 5 min at 500 g. Peptides were concentrated by speed-vac for 30 min at 45°C, or until ~2 μL remained. Samples were resuspended in 7 μL MS loading buffer (2% acetonitrile, 0.3% TFA) and injected in an Ultimate 3000 RSLCnano system (Thermo), separated in a 15-cm analytical column (75 μm ID home-packed with ReproSil-Pur C18-AQ 2.4 μm from Dr. Maisch) with a 50-min gradient from 3 to 16% acetonitrile in 0.1% formic acid followed by a 10-min gradient from 16 to 26% acetonitrile in 0.1% formic acid. The effluent from the HPLC was directly electrosprayed into a Q Exactive HF (Thermo) operated in data-dependent mode to automatically switch between full scan MS and MS/MS acquisition. Survey full scan MS spectra (from m/z 375–1600) were acquired with resolution 60,000 at m/z 400 (AGC target of 3 × 10^6^) and MS/MS spectra with resolution 15,000 at m/z 400 (AGC target of 1 × 10^5^). The 10 most intense peptide ions with charge states between 2 and 5 were sequentially isolated to a target value of 1 × 10^5^, and fragmented at 27% normalized collision energy. Typical MS conditions were: spray voltage, 1.5 kV; no sheath and auxiliary gas flow; heated capillary temperature, 250°C; ion selection threshold, 33.000 counts.

### Data analysis

Normalized heavy to light ratios reported by MaxQuant were log2 transformed for statistical analyses. We then selected the best 3 out of 4 paired replicates to reduce the number of dropouts caused by missing values. Missing value imputation was not performed. Differential abundance of peptides based on inhibitor administration was estimated by *t*-tests (with Welch correction, two sided, unpaired) on the log ratios of forward versus reverse labelled replicates. This approach avoids systematic errors based on arginine to proline conversion [41] leading to a systematic distortion of heavy/light ratios. MS data plots were prepared using Python libraries matplotlib, seaborn, and plotly. Sequence motif plots were prepared with WebLogo 3 [42]. Gene ontology analysis was done with DAVID 3.8 GOTERM_BP_DIRECT and proteins were further grouped into six categories based on the DAVID results [43, 44].

The mass spectrometry proteomics data and the data supplement have been deposited to the ProteomeXchange Consortium via the PRIDE [45] partner repository with the dataset identifier PXD014825 at https://www.ebi.ac.uk/pride/archive/.

## SUPPLEMENTARY MATERIALS



## Abbreviations

CDK9: cyclin-dependent kinase 9
Pol II: RNA polymerase II
CTD: C-terminal domain
SILAC: stable isotope labeling with amino acids in cell culture
1-NA-PP1: 1-(1,1-dimethylethyl)-3-(1-naphthalenyl)-1H-pyrazolo[3,4-d]pyrimidin-4-amine
