# Supplementary material for: Analog-sensitive cell line identifies cellular substrates of CDK9
Source: Oncotarget. 2019 Dec 10;10(65):6934–43. doi: 10.18632/oncotarget.27334 (PMC6916755; doi:10.18632/oncotarget.27334)
Supplement: Supplementary file 1 [file oncotarget-10-6934-s001.pdf]

Analog-sensitive cell line identifies cellular substrates of CDK9

SUPPLEMENTARY MATERIALS

Note: The mass spectrometry proteomics data and the data supplement have been deposited to the ProteomeXchange Consortium via the PRIDE [1] partner repository with the dataset identifier PXD014825 at https://www.ebi.ac.uk/pride/archive/.

REFERENCES

1. Perez-Riverol Y, Csordas A, Bai J, Bernal-Llinares M, Hewapathirana S, Kundu DJ, Inuganti A, Griss J, Mayer G, Eisenacher M, Pérez E, Uszkoreit J, Pfeuffer J, et al. The PRIDE database and related tools and resources in 2019: improving support for quantification data. Nucleic Acids Res. 2019; 47:D442–D450. https://doi.org/10.1093/nar/gky1106. [PubMed]

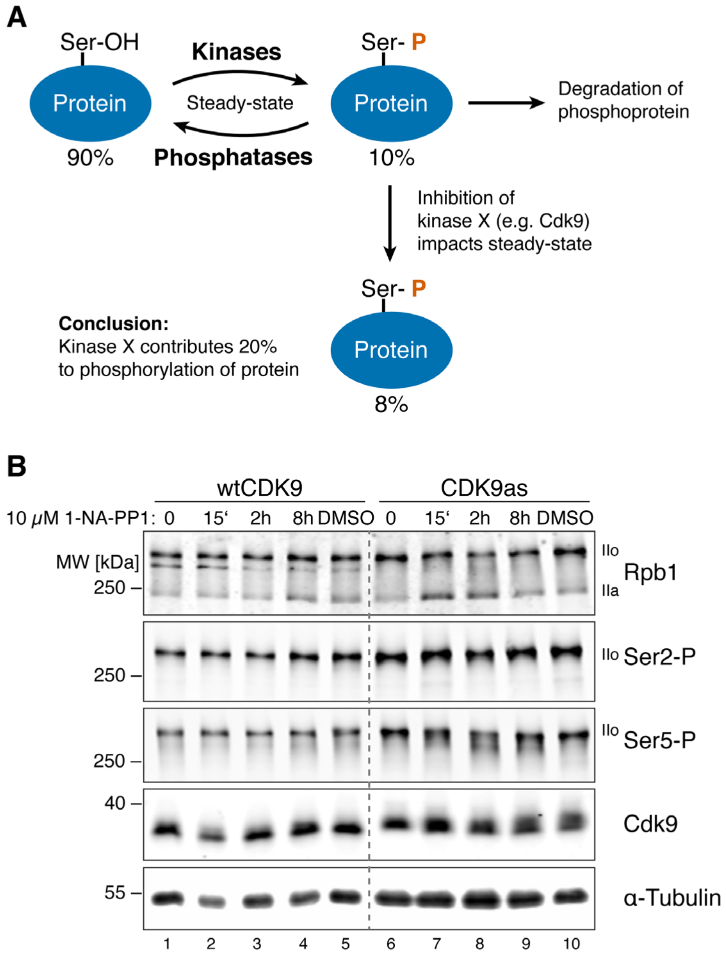

Supplementary Figure 1: (**A**) Kinases and phosphatases determine the steady-state of a given protein phosphorylation. Inhibition of a specific kinase and subsequent quantitation of the change in protein phosphorylation describes the contribution of the inhibited kinase to the steady-state. (**B**) Inhibition of CDK9as with 1-NA-PP1 reduces CTD phosphorylation signals. Wildtype (wt) CDK9 and CDK9as cells were treated with 10 μM 1-NA-PP1 for 15 min, 2 h, and 8 h. Untreated and 8 h DMSO-treated cells were used as controls. Phosphorylation levels of Pol II CTD were assessed by western blot using antibodies against Pol II large subunit Rpb1, CTD phosphorylated at Ser2 and Ser5. Cdk9 and α-Tubulin served as loading control.

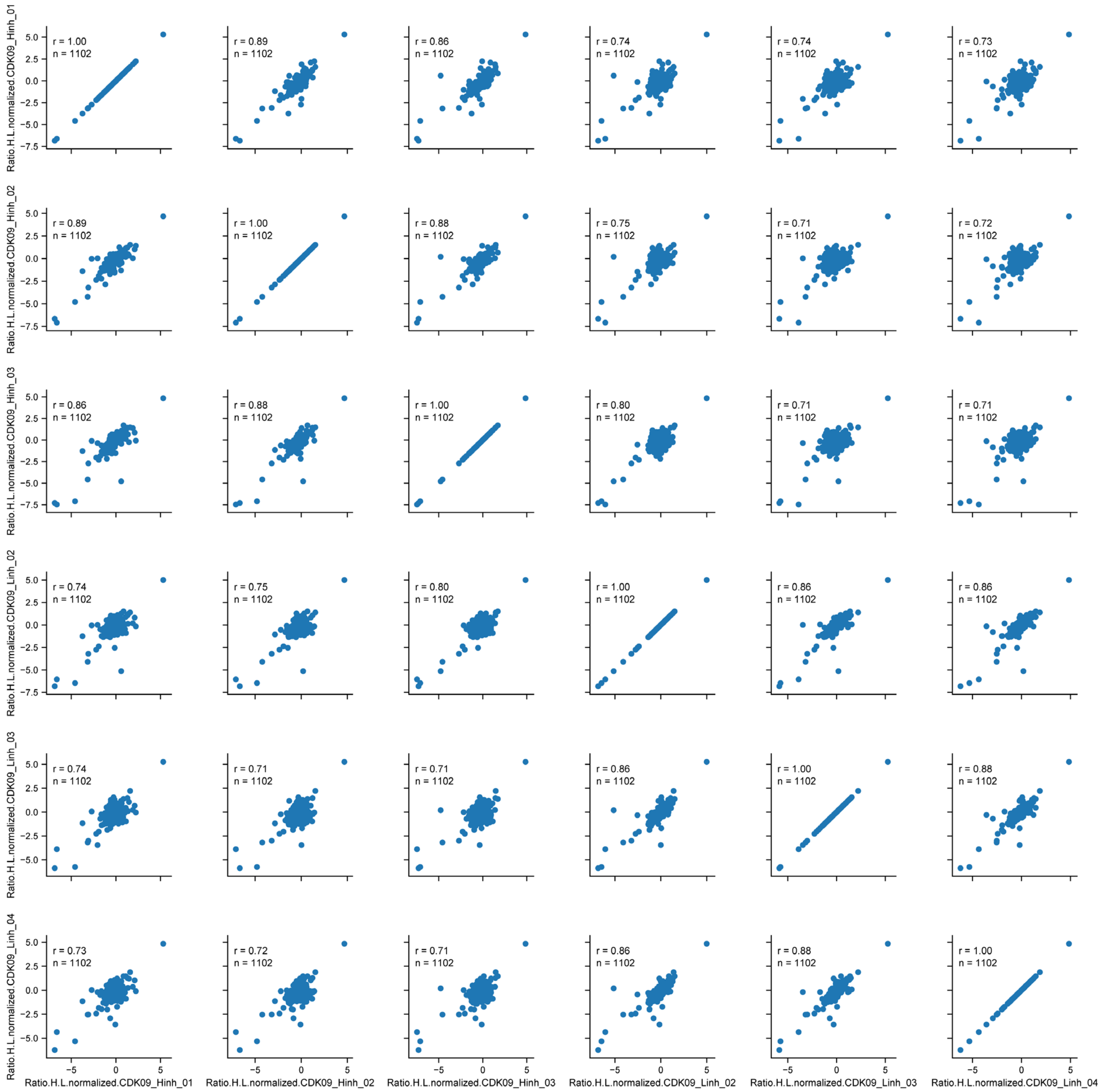

Supplementary Figure 2: Correlation of 1NA-PP1/DMSO ratios among all replicates was determined (r = Spearman correlation coefficient).

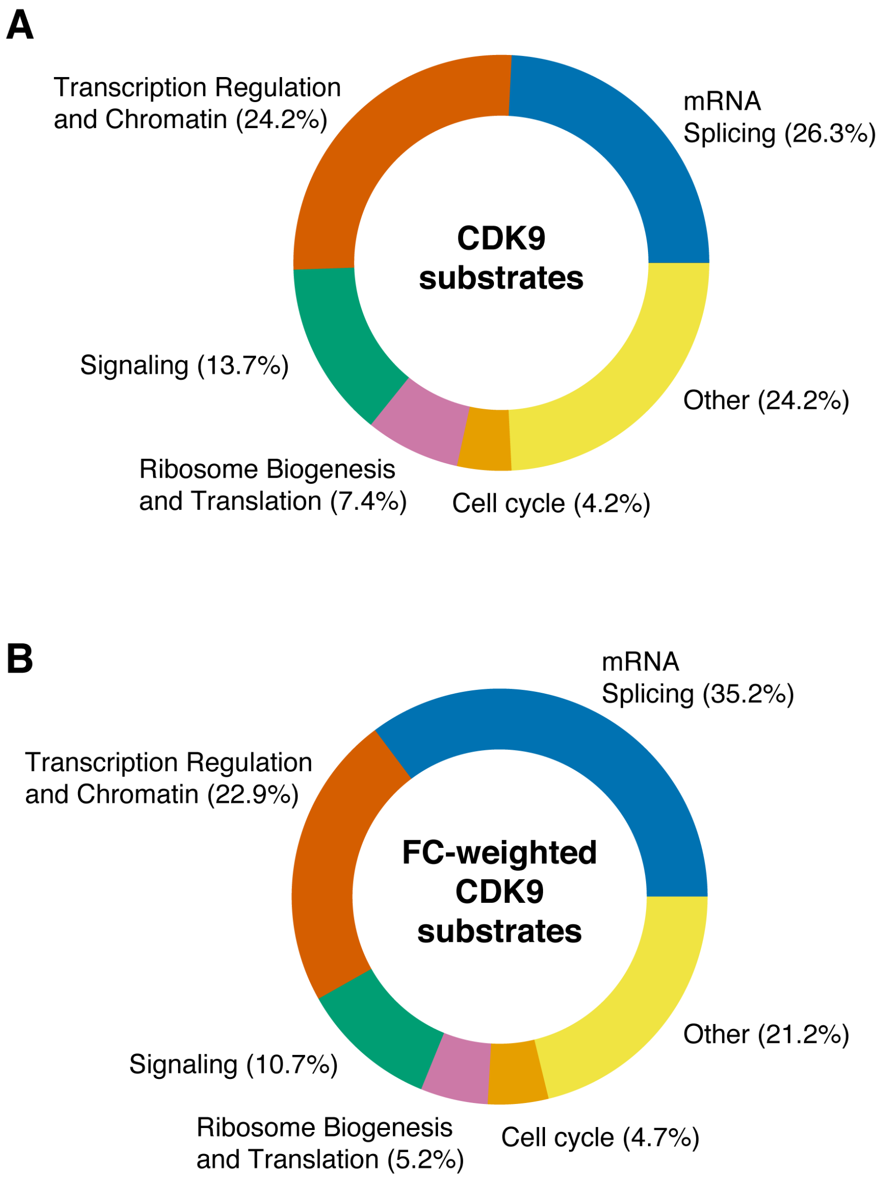

Supplementary Figure 3: Gene ontology analysis: (**A**) Based on number of substrates in each functional category. (**B**) Weighted gene ontology analysis, based on summed fold-change (FC) values of all substrates within one functional category.

Supplementary Table 1: Top 30 increased CDK9 substrates, ranked by log2(fold-change)

Name	Uniprot	Phosphosite	Log2(FC)	*P*-value
LEO1	Q8WVC0	S273, S277, S279	0.76	6.17E-05
LEO1	Q8WVC0	S277, S279	0.76	6.17E-05
RCSD1	Q6JBY9	S105, S108	0.76	0.0031
PPP1R9B	Q96SB3	S100	0.69	2.91E-07
MAPK14	E7EX54	T103, Y105	0.64	7.14E-05
PARN	O95453	S557	0.64	8.04E-05
DENND4A	Q7Z401	S1194	0.59	8.51E-05
DENND4A	Q7Z401	S1194, T1197	0.59	8.51E-05
EEF2	P13639	T57, T59	0.59	0.0124
WASF2	Q9Y6W5	S293, S296	0.58	0.000644
WASF2	Q9Y6W5	S296	0.58	0.000644
KLC1	Q07866	S521, S524	0.51	0.000996
UBXN7	C9JAT7	S118, S126	0.5	1.88E-05
TBC1D10B	Q4KMP7	S687, S707	0.5	0.0019
LRMP	F5H006	T266, S269	0.48	0.00641
MCRIP2	Q9BUT9	S82	0.47	0.000227
SRSF5	Q13243	S250	0.46	0.000958
RCSD1	Q6JBY9	S68	0.45	0.000676
UBXN7	C9JAT7	S118, S126	0.45	0.00207
BCLAF1	E9PK09	S264, S268	0.42	3.66E-06
PNN	Q9H307	S66	0.42	0.0222
LSM14A	Q8ND56	S178, S183	0.4	0.0048
SASH3	O75995	S38, S42	0.39	2.16E-05
SASH3	O75995	S38, S42	0.39	2.16E-05
STK4	F5H5B4	S355	0.37	0.00495
MYO9B	M0R300	S1267, T1271	0.35	0.00336
SCAMP3	O14828	S32	0.35	0.0116
CEP170	Q5SW79	S356, S359	0.34	0.00129
ARFGEF2	Q9Y6D5	S1511	0.33	0.00111
YBX3	P16989		0.33	0.0469
